# Recombinant *Lactobacillus plantarum* expressing and secreting heterologous oxalate decarboxylase prevents renal calcium oxalate stone deposition in experimental rats

**DOI:** 10.1186/s12929-014-0086-y

**Published:** 2014-08-30

**Authors:** Ponnusamy Sasikumar, Sivasamy Gomathi, Kolandaswamy Anbazhagan, Albert Abhishek, Eldho Paul, Varadaraj Vasudevan, Sundaresan Sasikumar, Govindan Sadasivam Selvam

**Affiliations:** Department of Biochemistry, Centre for Advanced Studies in Organismal and Functional Genomics, School of Biological Sciences, Madurai Kamaraj University, Madurai, 625 021 India; INSERM-U844, Insitut des Neuroscience de Montpellier Building, Hopital St. Eloi, 34091 Montpellier, France

**Keywords:** Calcium oxalate stone formation, Hyperoxaluria, Lactobacillus plantarum, Oxalate-degrading bacteria, Oxalate Decarboxylase, Urolithiasis

## Abstract

**Background:**

Calcium oxalate (CaOx) is the major constituent of about 75% of all urinary stone and the secondary hyperoxaluria is a primary risk factor. Current treatment options for the patients with hyperoxaluria and CaOx stone diseases are limited. Oxalate degrading bacteria might have beneficial effects on urinary oxalate excretion resulting from decreased intestinal oxalate concentration and absorption. Thus, the aim of the present study is to examine the *in vivo* oxalate degrading ability of genetically engineered *Lactobacillus plantarum* (*L. plantarum*) that constitutively expressing and secreting heterologous oxalate decarboxylase (OxdC) for prevention of CaOx stone formation in rats. The recombinants strain of *L. plantarum* that constitutively secreting (WCFS1OxdC) and non-secreting (NC8OxdC) OxdC has been developed by using expression vector pSIP401. The *in vivo* oxalate degradation ability for this recombinants strain was carried out in a male wistar albino rats. The group I control; groups II, III, IV and V rats were fed with 5% potassium oxalate diet and 14^th^ day onwards group II, III, IV and V were received esophageal gavage of *L. plantarum* WCFS1, WCFS1OxdC and NC8OxdC respectively for 2-week period. The urinary and serum biochemistry and histopathology of the kidney were carried out. The experimental data were analyzed using one-way ANOVA followed by Duncan’s multiple-range test.

**Results:**

Recombinants *L. plantarum* constitutively express and secretes the functional OxdC and could degrade the oxalate up to 70–77% under *in vitro*. The recombinant bacterial treated rats in groups IV and V showed significant reduction of urinary oxalate, calcium, uric acid, creatinine and serum uric acid, BUN/creatinine ratio compared to group II and III rats (*P* < 0.05). Oxalate levels in kidney homogenate of groups IV and V were showed significant reduction than group II and III rats (*P* < 0.05). Microscopic observations revealed a high score (4+) of CaOx crystal in kidneys of groups II and III, whereas no crystal in group IV and a lower score (1+) in group V.

**Conclusion:**

The present results indicate that artificial colonization of recombinant strain, WCFS1OxdC and NC8OxdC, capable of reduce urinary oxalate excretion and CaOx crystal deposition by increased intestinal oxalate degradation.

**Electronic supplementary material:**

The online version of this article (doi:10.1186/s12929-014-0086-y) contains supplementary material, which is available to authorized users.

## Background

The lifetime risk for kidney stone disease currently exceeds 6–12% in the general population, and its prevalence appears to increase steadily in both sexes [1]. Calcium oxalate (CaOx) is the major constituent of about 75% of all urinary stones population [2]. Secondary hyperoxaluria either based on intestinal hyperabsorption of oxalate or high intake of oxalate is considered a crucial risk factor in the pathogenesis of CaOx stone formation [3]. Urinary oxalate (UOx) is predominantly derived from endogenous production of oxalate from ingested or metabolically generated precursors and from the diet. It has been suggested that dietary contribution to UOx excretion is up to 50% [4]. Some foods, particularly vegetables such as spinach, wheat bran, and cereals contain high amounts of oxalic acid [5]. An increased absorption of oxalate has been demonstrated in 46% of patients with CaOx kidney stone [6]. Existing treatments for patients with CaOx urolithiasis are limited and do not always lead to sufficient reduction in UOx excretion. Even though, the invasive technologies (shockwave lithotripsy, ureteroscopy, percutaneous stone extractions) exist, these techniques have its own disadvantages like renal injury, recurrent stone formation with a prevalence of 50% over 10 years.

Another possible approach to prevent renal stone recurrence is to reduce the consumption of oxalate rich foods. Although, such dietary restriction is commonly advised to reduce stone recurrence, its long-term effectiveness is uncertain and would probably lead to deficiency in essential nutrients [7]. Thus, other methods meant to reduce intestinal oxalate absorption are required. Among them, the microbiological approach has received increasing attention in recent years. Oxalate degrading bacteria is being considered for degrading intestinal oxalate to prevent CaOx stone formation. Starting in 1980 with the discovery of an oxalotropic gut-resident bacterium *Oxalobacter formigenes* (*O. formigenes*) leading to a new research direction for the management of CaOx urolithiasis. *O. formigenes* is an anaerobic bacterium that naturally colonizes the colon of vertebrates, including humans, and utilizes oxalic acid as its sole source of energy [8]. The use of *O. formigenes* in reduction of oxalate excretion in urine and prevention of renal stone recurrence was elaborately studied [9,10]. However, endogenously derived oxalate supplement was needed to colonize the bacterium in the gut. Hence, usage of this bacterium raises some concern and the other side Oxalobacter strains are not considered mainstream therapy primarily due to lack of sufficient clinical data supporting their use. Earlier, reports have shown that lactic acid bacteria (LAB) have no influence on reduction of hyperoxaluria [11]. The discovery of oxalate decarboxylase (*oxdC*) gene in *Bacillus subtilis* (*B. subtilis*), which breaks down the oxalate in to formate and CO_2_ raise a new hope to mitigate hyperoxaluria [12]. In subsequent years various research groups have demonstrated the use of oxalate decarboxylase (OxdC) protein in degradation of oxalate by *in vitro* and *in vivo* experiment for the treatment of hyperoxaluria [13-15].

Hence, we designed a strategy to engineer LAB component of intestinal microflora by heterologous expression of *oxdC* gene from *B. subtilis* origin. Artificial colonization with this recombinant strain may decrease the intestinal oxalate absorption and renal excretion by degrading dietary oxalate. In the present work, *in vivo* oxalate degrading potency of two recombinants *Lactobacillus plantarum* (*L. plantarum*) strains such as OxdC-secretory WCFS1OxdC [16] and non-secretory NC8OxdC [17] was investigated in rats fed with oxalate-rich diet.

## Methods

### Chemicals and reagents

Primers used were synthesized and procured from Sigma Aldrich (USA) [Additional file 1]. The experimental diet containing 5% potassium oxalate was procured from National Institute of Nutrition (NIN, Hyderabad, India). Hyperoxaluria and calcium oxalate crystal were induced in a rat model as described elsewhere [18]. Urinary and serum biochemical parameters were measured in semi automated photometer 5010 V5 + (Robert Riele GmbH, Germany) using commercially available kits [Additional file 2].

### Bacterial strains, media and growth conditions

The bacterial strains and plasmids used in this study are listed in table 1. *L. plantarum* was grown in deMan-Rogosa-Sharpe (MRS) media at 30°C without shaking. Erythromycin was added to the MRS at a final concentration of 5 μg/mL for the growth of recombinant *L. plantarum*.

**Table 1:** **Bacterial strains and plasmids used in this work**

**Strains & plasmids**	**Characteristics** ^**α**^	**Source/references**
**Strains**		
*L. plantarum*		
WCFS1*	Host strain, Plasmid-free, silage isolate	Kleerebezem *et al.,* [19]
NC8OxdC	p256/pUC(pGEM)ori;P_*ldhL*_;*oxdC*;Erm^r^	Kolandasamy *et al.,* [17]
WCFS1OxdC	p256/pUC(pGEM)ori;P_*ldhL*;_sp_Lp_0373_ fused to the *oxdC*; Erm^r^	Sasikumar *et al.,* [16]
**Plasmid**		
pLdhlOxdC	p256/pUC(pGEM)ori;P_*ldhL*_;*oxdC*;Erm^r^	Kolandasamy *et al.,* [17]
pLdhl0373OxdC	p256/pUC(pGEM)ori;P_*ldhL*;_sp_Lp_0373_ fused to the *oxdC*; Erm^r^	Sasikumar *et al.,* [16]

^**α**^For strains, genotypic and phenotypic characteristics are given; for plasmid, plasmid and cloned-cassette characteristics are given; Erm^r^, : resistance to erythromycin.

*L. plantarum WCFS1 is a single colony isolate of strain NCIM8826 (Kleerebezem *et al.,* 2003) [19].

### Manipulation of recombinant *Lactobacillus plantarum*

The genetically engineered OxdC-secreting *L. plantarum* WCFS1OxdC was developed [16] and the construction of non-secreting *L. plantarum* NC8OxdC was described [17] and both the recombinants and non-recombinant *L. plantarum* WCFS1 strain was used to evaluate *in vivo* oxalate degradation in rat model.

### Preparation of live bacterial inocula

The recombinant WCFS1OxdC, NC8OxdC and the non-recombinant strain of *L. plantarum* WCFS1 was grown in MRS medium. The bacterial number per milliliter of cultures was estimated using spectrophotometric measurements (OD_600_) and cellular pellets were harvested by centrifugation at 5000 rpm. The pellet was washed and resuspended in sterile phosphate buffered saline (PBS) at (5X10^10^ CFU mL^−1^) [10].

### Animals and study design

Male wistar albino rats (130–140 g) were used in this study and the experimental procedure was approved by the Internal Research and Review Board, Ethical Clearance, Biosafety and Animal Welfare Committee of Madurai Kamaraj University. The rats were divided into five groups (n = 6/group) and were kept at 27 ± 2°C with a 12 h light and dark cycle. Group I control rats received standard rat chow and the experimental group rats (II, III, IV and V) received chow mixed with 5% potassium oxalate (weight/weight oxalate/chow) to induce hyperoxaluria [18]. The rats in group III, IV and V were orally administered with non-recombinant and recombinants *L. plantarum* respectively by esophageal gavage of (5X10^10^ CFU mL^−1^ day^−1^) bacterium [10]. Day 14 onwards the group II rats were administrated by esophageal gavage with 1 mL PBS day^−1^; while group III were administrated with non-recombinant *L. plantarum*; group IV and V rats were administrated with recombinant *L. plantarum* harboring plasmid pLdhl0373OxdC and pLdhlOxdC respectively. At the end of the fourth week, the animals were sacrificed and serum samples was separated. Kidney tissues were processed for localization of crystals, biochemical and various other morphological analyses.

### Urine collection and analysis

On the day 0, 7, 14, 21 and 28 the rats were placed in metabolic cages and 24 h urine was collected in presence of 0.02% sodium azide to prevent bacterial growth. After determining urinary volume and pH, urine was aliquot for various assays. Urinary oxalate, calcium, uric acid, creatinine and urea were also determined using commercial kit in semiautomatic photometer according to manufacturer’s protocol. Each week one-hour urine samples were collected and examined by polarized light microscopy to analyze the presence of CaOx crystalluria and scored on a basis of 0-3+ [20].

### Determination of recombinant *L. plantarum* in feces

Determination of recombinant *L. plantarum* in feces was carried out by culture methods as well as by PCR as described elsewhere [10].

### Serum parameters analysis

Serum parameters such as creatinine, calcium, urea, uric acid, protein and C -reactive protein (CRP) were measured by using respective kits as suggested by manufacturer (Additional file 2).

### Analysis of oxalate and calcium in kidney homogenate

A pair of kidney from each group rats was removed and a section of kidney was used for analysis of oxalate and calcium. Kidney tissue was rinsed with ice cold saline (0.9% w/v sodium chloride) and repeatedly washed with 0.15 M KCl, weighed, homogenized using 10% HCl and was centrifuged at 2500 rpm for 3 min. The supernatant was used to determine oxalate and calcium. Oxalate concentration was determined manually by colorimetric method described elsewhere [21].

### RNA isolation and semi-quantitative RT-PCR

The mRNA levels of glyceraldehyde-3 phosphate dehydrogenase (*GAPDH*), *OPN*, *renin*, and *ACE* in the kidney were quantified by semi-quantitative reverse transcriptase-polymerase chain reaction (RT-PCR). [Additional file 3].

### Analysis of histopathology and CaOx crystal in kidney

The kidney tissue from each group was fixed in 10% neutral buffered formalin, trimmed, processed, and embedded in paraffin. Sections from each kidney were stained with hematoxylin and eosin and examined under light microscope for pathological analysis and polarized light microscope for visualizing CaOx crystal. The presence of CaOx crystal was scored on a basis of 0-5**+** [22]. CaOx crystal present in each kidney tissue was examined by pizzolato staining methods [23]. Pathological analysis was examined with the help of qualified pathologist.

### Statistical analysis

Data were expressed as mean ± SD. The statistical significance between subgroups was analyzed with one-way ANOVA followed by Duncan’s multiple-range test using SPSS, software. Results were considered significant if the *P* value < 0.05.

## Results

### Engineered LAB efficiently degraded oxalate under *in vitro*

**Figure 1:**
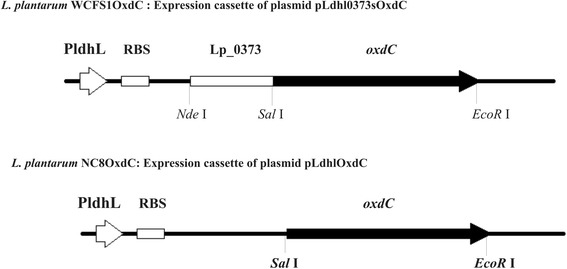
**Schematic representation of expression cassettes of recombinant plasmids.** *L. plantarum* WCFS1OxdC represents the recombinant strain harboring the plasmid pLdhl0373OxdC for extracellular expression of OxdC; *L. plantarum* NC8OxdC indicates the recombinant strain harboring the plasmid pLdhlOxdC for intracellular expression of OxdC; PldhL: promoter, RBS: ribosomal binding site; Lp_0373: signal peptides; *oxdC*: oxalate decarboxylase; restriction sites also indicated.

**Figure 2:**
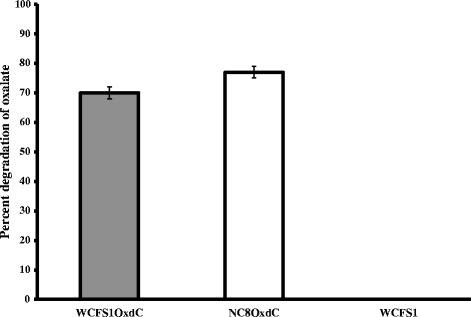
**Percentage of in vitro oxalate degradation by recombinant and wild type** ***L. plantarum*** **. **WCFS1OxdC: *L. plantarum* harboring the recombinant plasmid pLdhl0373OxdC; NC8OxdC: *L. plantarum* harboring recombinant plasmid pLdhlOxdC; WCFS1: wild type *L. plantarum*. The error bar represents the standard deviation from three independent exprements.

The recombinant OxdC-secretory *L. plantarum* WCFS1OxdC harboring the recombinant vector pLdhl0373OxdC size of 4.7 kb and non-secretory *L. plantarum* NC8OxdC harboring the recombinant plasmid without signal peptide sequence pLdhlOxdC was used to analyze *in vivo* oxalate degradation in rat model. Schematic representation of expression cassette of recombinant plasmids used for secretion and expression of OxdC in the *L. plantarum* was shown in Figure 1. The OxdC-secreting WCFS1OxdC strain harboring plasmid (pLdhl0373OxdC) was consisting of constitutive promoter (P_*ldhL*_) and signal peptide (Lp_0373) sequences, as a result the WCFS1OxdC strain secretes the functional OxdC at extracellular level and degrading 70% of extracellular oxalate (Figure 2). The specific activity of recombinant OxdC purified from recombinant strain of WCFS1OxdC was found to be 19.1 U/mg and secretion efficiency of the strain WCFS1OxdC shows that 25% of the OxdC produced was secreted into the medium. The OxdC non-secreting NC8OxdC strain which harboring recombinant plasmid (pLdhlOxdC), consisting of constitutive promoter (P_*ldhL*_) and lacking the signal peptide sequences. Thus, NC8OxdC strain expressing biologically active OxdC at intracellular level and degrading 77% of oxalate under *in vitro* condition (Figure 2). Whereas the wild type *L. plantarum* WCFS1 unable to degrade the oxalate as expected.

### Oxalate degrading recombinant LAB improved primary health of hyperoxaluric rat

Control rats (group I), received standard chow, and experimental rats (group II, III, IV and V), which received oxalate mixed food stayed healthy and gained weight. However with time, experimental rats gained significantly lesser weight than control (*P* < 0.05), while rats in groups IV and V receiving the recombinant *L. plantarum* WCFS1OxdC and NC8OxdC respectively gained more weight than groups II and III (*P* < 0.05, Table 2). Urinary pH was seen lower in experimental rats than control (*P* < 0.05, Table 2) and pH of group IV and V showed increased level than group II and III (*P* < 0.05).

**Table 2:** **Urinary biochemistry profile**

**Days** ^**α**^	**Group I (n = 6)**	**Group II**	**Group III**	**Group IV**	**Group V**
**Bodyweight (g)**					
**0**	140.40 ± 1.24	130.70 ± 3.18	131.01 ± 3.42	133.57 ± 2.66	133.38 ± 2.27
**7**	173.49 ± 2.34	134.37 ± 2.01^a*^	145.59 ± 2.82^a*^	142.27 ± 3.70^a*^	140.42 ± 2.10^a*^
**14**	218.31 ± 3.32	151.16 ± 2.64	160.88 ± 2.69	164.90 ± 3.38	150.82 ± 3.01
**21**	240.39 ± 2.75	171.17 ± 3.04	180.17 ± 3.25	190.76 ± 3.39	171.42 ± 3.35
**28**	261.11 ± 2.87	195.13 ± 3.70^a*^	191.80 ± 2.06^a*^	220.14 ± 2.68^a* b*c*^	201.73 ± 2.51^a* b*c*^
**Urine pH**					
**0**	7.07 ± 0.11	6.83 ± 0.26	6.84 ± 0.19	6.74 ± 0.25	6.54 ± 0.15
**7**	7.13 ± 0.16	7.08 ± 0.18	6.94 ± 0.20	7.11 ± 0.18	7.29 ± 0.12
**14**	7.10 ± 0.22	6.94 ± 0.30	6.94 ± 0.24	6.97 ± 0.20	7.02 ± 0.15
**21**	7.21 ± 0.15	6.44 ± 0.19^a*^	6.55 ± 0.12^a*^	6.86 ± 0.21^b*c*^	6.94 ± 0.16
**28**	7.25 ± 0.11	6.09 ± 0.07^a*^	6.16 ± 0.08^a*^	6.90 ± 0.17^a* b*c*^	6.79 ± 0.13^a* b*c*^
**Uric acid (mg/24 h)**					
**0**	0.05 ± 0.01	0.09 ± 0.01	0.06 ± 0.01	0.05 ± 0.01	0.05 ± 0.01
**7**	0.12 ± 0.01	0.14 ± 0.01	0.16 ± 0.01	0.07 ± 0.01	0.12 ± 0.04
**14**	0.11 ± 0.02	0.18 ± 0.01^a*^	0.19 ± 0.01^a*^	0.09 ± 0.01^b*c*^	0.14 ± 0.01^b*c*^
**21**	0.15 ± 0.02	0.21 ± 0.02^a*^	0.25 ± 0.03^a*^	0.11 ± 0.03^b*c*^	0.15 ± 0.01^b*c*^
**28**	0.17 ± 0.02	0.46 ± 0.02^a*^	0.39 ± 0.03^a*^	0.12 ± 0.01^b*c*^	0.18 ± 0.02^b*c*^
**Creatinine (mg/24 h)**					
**0**	1.33 ± 0.08	1.39 ± 0.18	1.23 ± 0.15	1.11 ± 0.11	1.16 ± 0.08
**7**	1.04 ± 0.06	1.50 ± 0.16^a*^	1.69 ± 0.26^a*^	1.41 ± 0.11^a*^	1.73 ± 0.10^a*^
**14**	1.64 ± 0.24	1.73 ± 0.13^a*^	2.82 ± 0.29^a*^	1.61 ± 0.18^c*^	1.95 ± 0.18^c*^
**21**	1.51 ± 0.31	2.71 ± 0.19^a*^	3.19 ± 0.22^a*^	2.07 ± 0.09^a*b*c*^	2.30 ± 0.16^a*b*c*^
**28**	1.77 ± 0.23	3.69 ± 0.30^a*^	3.52 ± 0.19^a*^	2.52 ± 0.14^a*b*c*^	3.07 ± 0.61^a*c*^

^**α**^ Data are expressed as mean ± SD. Comparisons are made against Group I (Control)^a^ , Group II (lithiatic control)^b^ and Group III (Non-recombinant strain)^c^.

^a*^ ^b*^ and ^c*^ indicates the mean value is significant at p < 0.05 against group I, II and III correspondingly. n = 6 rats each group.

Urinary excretion of creatinine increased with time in all animals but it was significantly higher in experimental group than control (*P* < 0.05). However, at the end of experiment (Day 28), mean value of creatinine in groups IV and V showed significantly lower (*P* < 0.05) against group II and III rats (Table 2). Excretion of uric acid in groups II and III rats showed significant increase (*P* < 0.05) when compared to group I, IV and V (Table 2).

### Rats artificially colonized by recombinant LAB reduced urinary oxalate excretion

**Figure 3:**
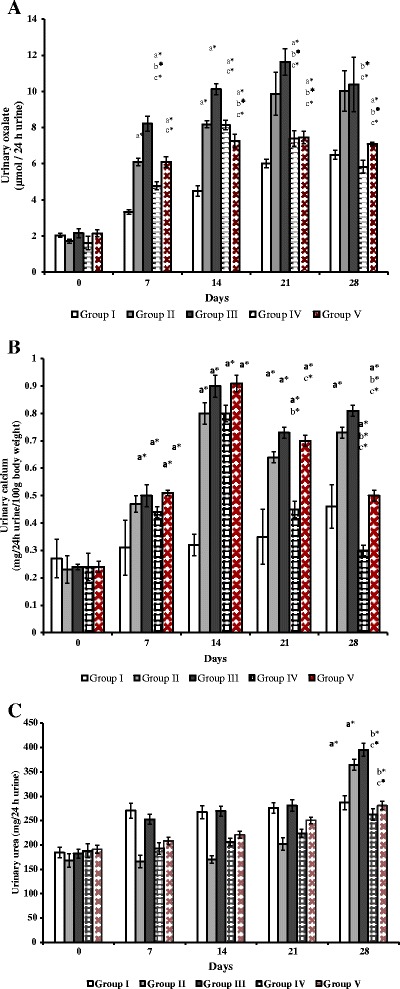
**Urinary oxalate, calcium and urea excretions in control and experimental rats. (A)** Urinary oxalate excretions in control and experimental rats **(B)** Urinary calcium excretions in control and experimental rats **(C)** Urinary urea concentrations in control and experimental rats. Comparisons are made against group I (Control)^a^, group II (lithiatic control)^b^ and group III (Non-recombinant strain)^c^. * The mean value is significant at p < 0.05. n = 6 rats each group.

Compared to baseline values of urinary oxalate (UOx), the excretion was significantly increased in all groups (*P* < 0.05). By days 7, 14, 21 and 28, excretion of urinary oxalate in groups II, III and V showed significantly increased level than group I (*P* < 0.05). On the other hand, the excretion of oxalate in group IV rats showed significant variations on day 7, 14 and 21 when compared to group I (*P* < 0.05), whereas, on 28^th^ day no significant variation was observed (Figure 3A). When the comparisons were made between group II and treated groups (III, IV and V) the UOx excretion on day 21 and 28, groups IV and V rats showed significant reduction than group II (*P* < 0.05). Similarly, when compared to non-recombinant bacterial treated group III, significant decrease of UOx excretion was seen in groups IV and V (*P* < 0.05), at the end of experiment (Figure 3A).

Urinary calcium on baseline does not show any significant change in all groups. Compared to the group I rats calcium level was increased significantly in all groups during the experimental days (*P* < 0.05). While compared to group II and III, the urinary calcium level dropped significantly in group IV on 21^st^ and 28^th^ day (*P* < 0.05), and group V shows significantly lower level against group II and III rats at 28^th^ day (*P* < 0.05, Figure 3B). Urea level of all groups at baseline, 7^th^, 14^th^ and 21^st^ day did not show any significant difference against group I, whereas on 28^th^ day the group II and III showed significantly increased level than group I rats (*P* < 0.05). On the other hand, significantly decreased level of urea was observed in groups IV and V against groups II and III (*P* < 0.05, Figure 3C).

### Recombinant *L. plantarum* survived in rat intestine

**Figure 4:**
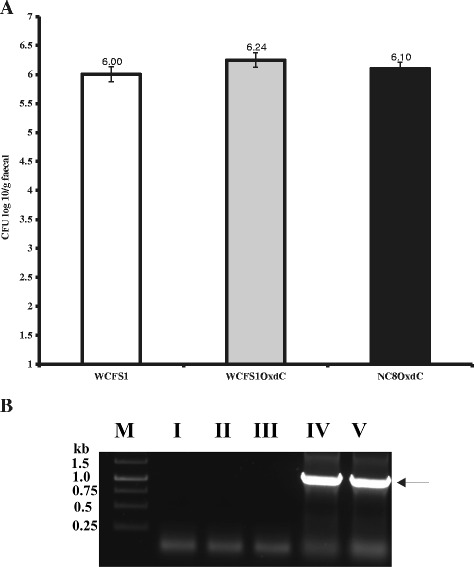
**Colony forming units of wild type and recombinant** ***L. plantarum*** **in rat feces. (A)** WCFS1 indicate wild type *L. plantarum* and WCFS1OxdC, NC8OxdC indicate recombinant *L. plantarum* harboring plasmid pLdhl0373OxdC and pLdhlOxdC respectively. Results are expressed as mean of colony forming units (CFU) per gram of feces **(B)** Determination of recombinant *L. plantarum* in feces by PCR. I, II, III, IV and V indicate the respective group of rat, M; 1 kb DNA Marker, Arrow indicate the PCR amplicon corresponding to 1.2 kb size of *oxdC* gene.

The colony forming units (CFU) method and PCR was used to detect the presence of live recombinant and non-recombinant *L. plantarum* in the intestine of treated rats. Mean colony forming units (CFU) per gram of feces in group III, IV and V was 6.00 ± 0.13 (*L. plantarum* WCFS1), 6.24 ± 0.12 (WCFS1OxdC) and 6.10 ± 0.10 (NC8OxdC) respectively (Figure 4A). Whereas, no strains were detected in the feces of groups I and II. PCR confirmed that the fecal DNA in group IV and V rats alone produces the amplicon corresponding to OxdC gene (1.2 kb) (Figure 4B).

### Prevention of crystalluria in recombinant treated rats

**Figure 5:**
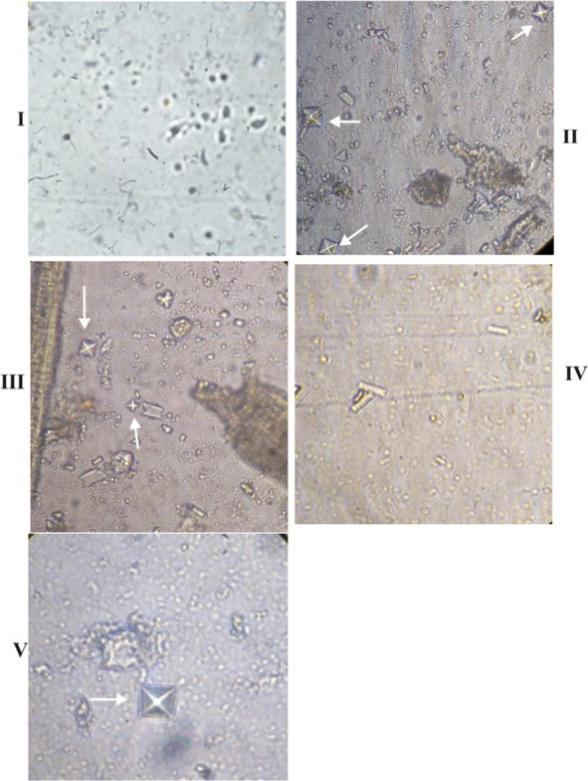
**Microscopy examinations of CaOx crystals in experimental rat urine at 20X magnification.** I, II, III, IV and V represent the respective group of rat. Arrow indicates the CaOx crystals urine sample of respective group rats.

All experimental rats were examined for the presence of CaOx crystal in urine after the administration of non-recombinant and recombinant *L. plantarum*. Group I control rats urine was devoid of any CaOx crystal throughout experimental period. By day 28, rats in groups II and III showed high score (2+) of CaOx crystal, while group V urine shows low score (1+). The group IV rats did not show any CaOx crystal (Figure 5).

### Recombinant *L. plantarum* maintained normal serum parameters in hyperoxaluric rats

Blood urea nitrogen and creatinine ratio (BUN/Creatinine) was calculated to predict the renal function. The mean value of BUN/Creatinine ratio in groups II and III rats was 41.04 ± 1.68 and 40.04 ± 0.54 respectively, against group I (37.52 ± 1.30). Whereas groups IV and V showed 34.61 ± 1.46 and 36.35 ± 1.19, which clearly reveal the significant difference in group II and III (*P* < 0.05) than group I. The uric acid was predicted to be increased in groups II and III against group I (*P* < 0.05). However, no significant difference was observed in groups IV and V against group I (Table 3). In order to predict the inflammation, C-reactive protein (CRP) level was measured in the serum sample of all groups. When compared to control group, significantly increased level of CRP was observed in experimental groups. The serum protein level of experimental groups (II, III, IV and V) showed significant decrease against control (*P* < 0.05, Table 3).

**Table 3:** **Serum profile**

**Parameters** ^**α**^	**Group I (n = 6)**	**Group II**	**Group III**	**Group IV**	**Group V**
**Total Protein (mg/dl)**	7.71 ± 0.45	6.24 ± 0.49^a*^	6.63 ± 0.48^a*^	6.34 ± 0.33^a*^	6.46 ± 0.41^a*^
**Uric acid (mg/dl)**	4.52 ± 0.33	6.61 ± 0.15^a*^	5.91 ± 0.25^a*^	4.35 ± 0.22^b* c*^	5.06 ± 0.35^b* c*^
**Calcium (mg/dl)**	12.46 ± 1.27	11.38 ± 1.18	10.95 ± 1.25	10.83 ± 0.92	10.81 ± 1.36
**BUN/ Creatinine ratio**	37.52 ± 1.30	41.04 ± 1.68^a*^	40.04 ± 0.54^a*^	34.61 ± 1.46^b* c*^	36.35 ± 1.19^b* c*^
**CRP (μg/ml)**	43.35 ± 2.18	59.72 ± 2.49^a*^	61.92 ± 2.37^a*^	50.75 ± 2.01^a* b* c*^	53.73 ± 2.74^a* b* c*^

^**α**^ Data are expressed as mean ± SD. Comparisons are made against Group I (Control)^a^ , Group II (lithiatic control)^b^ and Group III (Non-recombinant strain)^c^.

^a*^ ^b*^ and ^c*^ indicates the mean value is significant at p < 0.05 against group I, II and III correspondingly. n = 6 rats each group.

### Recombinant *L. plantarum* administered rats reduced oxalate level in kidney

**Figure 6:**
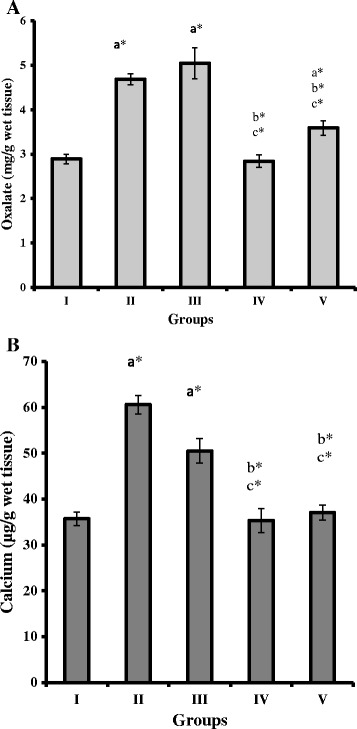
**Oxalate and calcium concentrations in kidney of control and experimental rats. (A)** Oxalate concentrations in the kidney of control and experimental rats (n = 6 rats per group). **(B)** Calcium concentrations in the kidney of control and experimental rats (n = 6 rats per group). Comparisons are made against Group I (Control)^a^, Group II (lithiatic control)^b^ and Group III (Non-recombinant strain)^c^. * The mean value is significant at p < 0.05.

Oxalate concentration in kidney tissue homogenate of groups II, III and V showed significant increase (*P* < 0.05) when compared to groups I and IV rats. However, the recombinant *L. plantarum* administered groups IV and V showed significantly decreased level of oxalate compared to group II and III (*P* < 0.05, Figure 6A). The concentration of calcium level significantly increased in groups II and III against groups I, IV and V rats (*P* < 0.05, Figure 6B).

### Gene expression analysis and renal histopathology revealed reversal of kidney stone-induced damage in hyperoxaluric rats

**Figure 7:**
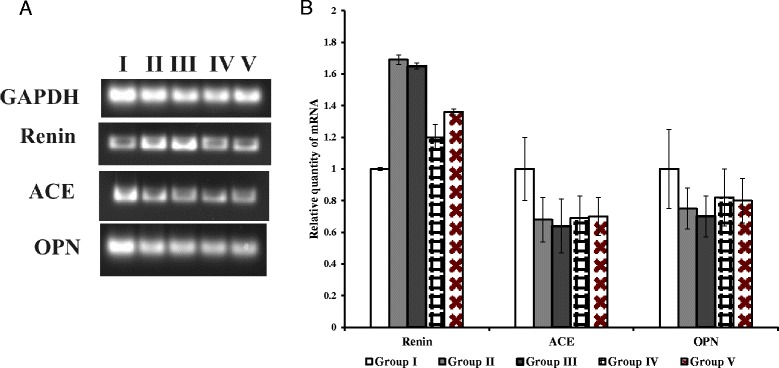
**Gene expression analysis using semi-quantitative RT-PCR. (A)** Semi–quantitative RT-PCR for quantification of *renin, ACE* and *OPN* mRNA in respective rats kidney tissue. The ethidium bromide stained gels were scanned using Bio-Rad Gel Doc XR and the intensity of PCR product was quantified using Image Lab Software version 5 (Bio-Rad). The final band intensity for OPN, ACE and renin were expressed relative to the reference gene GAPDH. The expression levels in the control group were considered the basal levels, and the others are expressed as fold change from the control group. **(B)** The fold change values represent the means ± S.E.M. of (n = 6) in the bar diagram. I, II, III, IV and V represent the respective rat groups.

**Figure 8:**
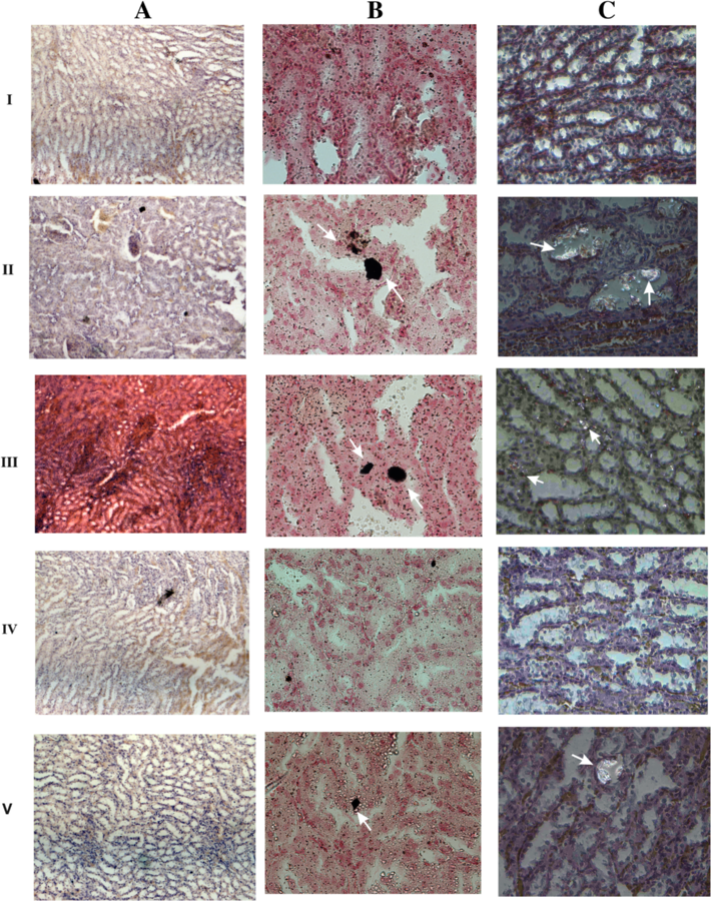
**Microscopy examinations of kidney tissue and CaOx crystals in experimental rat at 20X magnification.** I, II, III, IV and V represent the respective group of rat. **A**, **B** and **C** represents H&E stained section, pizzolato methods stained section for CaOx crystal and polarized microscopy examination of CaOx crystal respectively. Arrow indicates the CaOx crystal in kidney section of respective group.

Renal function was examined by using semi-quantitative PCR for *renin, ACE* and *OPN* expression. The up-regulation of renin mRNA was observed in groups II and III when compared to group I rats. While the recombinant bacterial treated group IV and V shows significant reduction in mRNA level compared to group II and III. The down regulations of ACE, OPN mRNA were seen in groups II, III, IV and V rats (Figure 7A, B). Histopathological examination of kidney sections of group I rats showed normal histological structures. Group II and III rats showed a reduced number of glomeruli and large areas of red blood cell casts with dialated tubules. Stroma showed hemorrhage and blood vessels were congested and thickened. Sections obtained from rats in the group IV administered with WCFS1OxdC revealed normal glomeruli with no red blood cast, but slight tubular necrosis. Examination of stroma shows areas of hemorrhage. Similarly, group V rats that received NC8OxdC showed normal glomeruli, but high tubular necrosis and congested blood vessels. The CaOx crystals were examined by pizzolato staining and also by using polarized microscopy. It revealed no incidence of CaOx crystal deposition in group I whereas as high score (4+) of CaOx crystals in groups II and III rats. However, group IV showed no identifiable crystal deposits in the kidneys and group V showed significantly lower score (1+) (Figure 8).

## Discussion

Dietary oxalate is a major contributor to urinary oxalate (UOx) excretion in humans [4]. The identification of intestinal oxalate degrading bacteria provided a new direction for the reduction of UOx [24]. The present study is to examine the efficacy of heterologous OxdC expressing and secreting recombinant *L. plantarum* to degrade the intestinal oxalate thereby preventing hyperoxaluria and CaOx urolithiasis in rats.

Previously, we reported *in vitro* degradation of oxalate by recombinant *L. plantarum* expressing heterologous OxdC at intracellular level [17]. Since the expression was intracellular, we made an attempt to express OxdC extracellularly to increase the oxalate degradation efficiency. Sasikumar *et al.* [25] analyzed the two homologous signal peptide (SP) such as Lp_0373 and Lp_3050 of *L. plantarum* for the extracellular expression OxdC under inducible condition and results shown that the SP (Lp_0373) efficiently secrete the OxdC than the SP (Lp_3050). Later on, by using previously characterized homologous promoter (P_ldhL_) and signal peptide (Lp_0373) sequences, the genetically modified constitutively OxdC-secretory WCFS1OxdC strain was developed [16]. The resulting *L. plantarum* strain found to be very efficient for secretion of OxdC and degradation of extracellular oxalate. Here, the intragastric oxalate degrading efficiency of intracellular and extracellular OxdC expressing recombinant *L. plantarum* was evaluated in rats. Results of plasmid segregation analysis reveal daily administration of recombinant *L. plantarum* is vital since the *L. plantarum* lost almost 70–90% of erythromycin-based plasmid [16]. Hence, artificial intestinal colonization and oxalate degradation in rat was established via the daily load, as a result the expression of OxdC was retained. In future, the plasmid can be stabilized by constructing mutants lacking essential genes like *alr* (alanine racemase), which can be complimented by adding back *via* the plasmid [26].

*O. formigenes* is efficient in oxalate degradation and had been proposed for its application for degrading intestinal oxalate [10,27,28]. Numerous studies have linked the absence of *O. formigenes* to higher UOx excretion [29,30]. Reports revealed no significant difference in UOx excretion between patients who tested positive or negative for *O. formigenes* [31]. In addition, colonization of *O. formigenes* in the gut require oral oxalate supplements [9]. Sidhu *et al.* [27] demonstrated that when oxalate is removed from the diet, artificially colonized rats lose colonization within 5 days. Since the uses of *O. formigenes* in mitigation of intestinal oxalate have difficulty, here we tried alternatively by using recombinant *L. plantarum* secreting OxdC protein extracellular level for degradation of intestinal oxalate.

The significant reduction of urinary oxalate excretion in group IV and V rats clearly illustrates the degradation of dietary oxalate by the presence of recombinant *L. plantarum* WCFS1OxdC and NC8OxdC. Hyperoxaluric conditions were observed in the absence of recombinant strain in group II and III rats. Even though, groups IV and V rats showed significant reduction in UOx excretion, the higher reduction was seen in group IV (43%) than in group V (30%) which suggested that intestinal oxalate in group IV is better degraded than in group V rats.

When compared to group II, 40% and 25% of total oxalate concentration was reduced in the kidney tissue of group IV and V rats and 45% and 30% of oxalate reduction when compared to wild type *L. plantarum* treated group III rats respectively. The higher reduction of oxalate in kidney tissue of group IV rats administrated with recombinant WCFS1OxdC strain was associated with the secretion of OxdC, which prevented hyperoxaluria effectively compared to non-secretory NC8OxdC strain treated rats (group V) by promoting higher degradation of intestinal oxalate. Increase in calcium and oxalate content in the renal tissue of group II and III were associated with oxalate supplemented diet. Orally administered *Escherichia coli* (*E. coli*) expressed recombinant *B. subtilis* OxdC has substantially declined the UOx level in experimental rat [13]. Oral therapy with crystalline, cross-linked formulation of the OxdC in mice diminishes symptoms of hyperoxaluria and urolithiasis [14]. Furthermore, orally given formulation of *B. subtilis* OxdC, was shown to be safe in rats and dogs during short-term toxicity tests [15]. Although, the use of OxdC enzyme to decompose intestinal oxalate was broadly demonstrated, this approach to treat hyperoxaluria can be very expensive and daily load of OxdC was also required. The recombinant *L. plantarum* developed in this study was degrading intestinal oxalate by simply colonizing bacterium in the gut. However, improvement in strategy of artificial colonization of the strain for its use as probiotics is majorly required.

The significantly lower excretion of urinary urea, uric acid, creatinine and serum BUN/Creatinine ratio, uric acid in recombinant strain administered rats in group IV and V reveals the oxalate mediated renal damage was protected in rats group by degrading intestinal oxalate and thereby preventing oxalate toxicity. Increased level of urinary creatinine and serum BUN/Creatinine ratio in group II and III rats associated with renal tissue damage and functional abnormalities by the oxalate induced toxicity. The changes in the urinary pH of rats in group II and III might be associated with the distal tubular dysfunction.

A significant increase in the expression of renin mRNA in kidneys of groups II and III rats suggesting higher oxalate stress in kidney due to the oxalate diet. While, reversed expression of renin mRNA in group IV and V indicating that oxalate stress in the kidney was reduced due to the degradation of oxalate in intestine by the administered recombinant *L. plantarum.* Similarly, the increase in renin mRNA expression is associated with hyperoxaluria and CaOx crystal deposition [32].

Microscopic examination of urinary sediments of oxalate-diet fed rats in groups II and III showed a high score of CaOx crystal than rats in groups IV and V at the end of experimental period. Earlier reports also suggested that administration of oxalate supplemented diet induced CaOx crystal in urine [33]. Polarized microscopic examination of paraffin kidney sections revealed no significant CaOx crystal in group IV rats that received OxdC-secreting strain (WCFS1OxdC), whereas, group V rats administered with non-secretory strain (NC8OxdC) showed lower CaOx crystal deposition. This observation reveal that kidney of group IV rats was better protected from oxalate toxicity compared to group V. But, group III rats receiving wild type *L. plantarum* showed higher crystal score, suggesting that the wild type strain does not degrade the intestinal oxalate that lead to higher crystal aggregation. Similar results were also observed in pizzolato stained kidney sections of experimental rat groups (I, II, III, IV and V). Histopathology observation of kidney tissue of groups II and III rats showed kidney damage, while the group IV and V rats kidney showed normal glomeruli with moderate and high necrosis respectively. The increased level of CRP in the serum of group II and III rats was associated with the renal inflammation and renal function abnormalities, which was also clearly observed in histological studies. However, the significantly decreased CRP levels were observed in groups IV and V compared to groups II and III rats, that indicates renal damage was protected due to the reduction of oxalate toxicity by the recombinant *L. plantarum*.

The present study showed the artificial colonization of *L. plantarum* harboring the plasmid pLdhl0373OxdC and pLdhlOxdC containing oxalate degrading gene (*oxdC*) decrease urinary oxalate excretion and CaOx crystal deposition in rats due to the degradation of dietary oxalate in intestine by OxdC expressing and secreting recombinant *L. plantarum*. However, using them as a probiotic require improvement by stabilizing the plasmid by constructing mutant strain lacking essential genes (eg., *thyA* or *alr)*.

## Conclusion

In conclusion, the current study indicate that daily oral administration of OxdC secretory *L. plantarum* WCFS1OxdC in rats associated with decreased excretion of urinary oxalate and reduced risk of calcium oxalate crystal formation. The results provide an evidence of colonization with recombinant *L. plantarum* capable of reducing urinary oxalate excretion which reflects increased intestinal oxalate degradation, leaving less oxalate available for absorption. Further, the findings of the above study help to develop a biologically contained recombinant bacterium with food-grade selection marker, used as a probiotic for the treatment of hyperoxaluria and calcium oxalate stone disease.

## Additional files

Additional file 1: Table S1. Primers used in this work. Additional file 2: Table S2. Kits used for biochemical parameters analysis. Additional file 3:
**RNA isolation and Semi – Quantitative RT-PCR.**

## Abbreviations

CaOx: Calcium Oxalate
UOx: Urinary Oxalate
*L. plantarum*: *Lactobacillus plantarum*
*O. formigenes*: *Oxalobacter formigenes*
LAB: Lactic acid bacteria
OxdC: Oxalate decarboxylase
SP: Signal Peptide
