# Supplementary material for: Recombinant Lactobacillus plantarum expressing and secreting heterologous oxalate decarboxylase prevents renal calcium oxalate stone deposition in experimental rats
Source: J Biomed Sci. 2014 Aug 30;21(1):86. doi: 10.1186/s12929-014-0086-y (PMC4256919; doi:10.1186/s12929-014-0086-y)
Supplement: Additional file 1: Table S1. — Primers used in this work. [file 12929_2014_86_MOESM1_ESM.docx]

**Additional file 1**

**Table S1 : Primers used in this work**

| **Primers*** | **Sequence (5ˈ→ 3ˈ) ^a^** |
| --- | --- |
| **OXDC - F** | 5ˈGAGAGTCGACATGAAAAAACAAAATGACATTCCGC3ˈ |
| **OXDC - R** | 5ˈGGAATTCGTGGTGGTGGTGGTGGTGTTATTTACTGCATTTCTTTTTTCACTAC3ˈ |
| **GAPDH – F** | 5ˈTGCCAAGTATGATGACATCAAGAA3ˈ |
| **GAPDH – R** | 5ˈAGCCCAGGATGCCCTTTAGT3ˈ |
| **OPN – F** | 5ˈTGAGACTGGCAGTGGTTTGC3ˈ |
| **OPN – R** | 5ˈCCACTTTCACCGGGAGACA3ˈ |
| **Renin – F** | 5ˈACCAGGGCAACTTTCACTACGT3ˈ |
| **Renin – R** | 5ˈACCCCCTTCATGGTGATCTG3ˈ |
| **ACE – F** | 5ˈTTGTCTGTCACTGGAGCCTGAT3ˈ |
| **ACE – R** | 5ˈCACACCCAAAGCAATTCTTCGT3ˈ |

^a^ The restriction sites are underlined; ***** F, R: represent the forward and reverse primer respectively
