# Supplementary material for: Recombinant Lactobacillus plantarum expressing and secreting heterologous oxalate decarboxylase prevents renal calcium oxalate stone deposition in experimental rats
Source: J Biomed Sci. 2014 Aug 30;21(1):86. doi: 10.1186/s12929-014-0086-y (PMC4256919; doi:10.1186/s12929-014-0086-y)
Supplement: Additional file 2: Table S2. — Kits used for biochemical parameters analysis. [file 12929_2014_86_MOESM2_ESM.docx]

**Additional file 2**

**Table S2: Kits used for biochemical parameters analysis.**

| S. No | Parameter | Manufacturers |
| --- | --- | --- |
| 1 | Total protein | Reckon Diagnostics Pvt., Ltd, India |
| 2 | Calcium (Arsenazo III method) | Reckon Diagnostics Pvt., Ltd, India |
| 3 | Creatinine | Piraml health care Pvt., Ltd, India |
| 4 | Uric acid | Piraml health care Pvt., Ltd, India |
| 5 | Urea | Piraml health care Pvt., Ltd, India |
| 6 | Oxalate (Oxalate assay method) | Trinity Biotech, Ireland |
