# Supplementary material for: Recombinant Lactobacillus plantarum expressing and secreting heterologous oxalate decarboxylase prevents renal calcium oxalate stone deposition in experimental rats
Source: J Biomed Sci. 2014 Aug 30;21(1):86. doi: 10.1186/s12929-014-0086-y (PMC4256919; doi:10.1186/s12929-014-0086-y)
Supplement: Additional file 3: — RNA isolation and Semi – Quantitative RT-PCR. [file 12929_2014_86_MOESM3_ESM.docx]

**Additional file 3**

**RNA isolation and Semi – Quantitative RT-PCR**

Briefly, total RNA was isolated from kidney using TRI reagent (Sigma Aldrich, USA) according to the manufacturer’s protocol. Two micrograms of total RNA was reverse-transcribed to cDNA. In brief, 50 µL reactions contained 3 µL of 100 mM MgCl2, 1.25 µL of RNase inhibitor, 5 µL of 10X PCR buffer, 10 µL of 10 mM dNTP mix, 1.3 µL of Oligo d(T) (Fermentas), and 1.5 µL of reverse transcriptase (Fermentas). This mixture was incubated 60 min at 37 °C, and then reaction mixture was heated to 94 °C for 5 min to stop the reaction. Primers used are listed in [see Additional file 1]. All PCR reactions were performed as descried [Umekawa et al 2004] using a GeneAmp PCR system 2700 (Applied Biosystem, USA). Finally the PCR products were resolved by electrophoresis through 2 % agarose gels along with 100bp DNA ladder. The ethidium bromide stained gels were scanned using Bio-Rad Gel Doc XR and the intensity of PCR product was quantified using Image Lab Software version 5 (Bio-Rad). The final band intensity for OPN, ACE and renin were expressed relative to the reference gene GAPDH.

**Reference**

Umekawa T, Hatanaka Y, Kurita T, Khan SR. Effect of angiotensin II receptor blockage on osteopontin expression and calcium oxalate crystal deposition in rat kidneys. J Am Soc Nephrol. 2004 Vol;15(3):635-44.
